# Synthesized Image Reconstruction for Post-Reconstruction Resolution Recovery

**DOI:** 10.1109/TRPMS.2023.3247489

**Published:** 2023-02-22

**Authors:** Laurence Vass, Andrew J. Reader

**Affiliations:** School of Biomedical Engineering and Imaging SciencesKing’s College London WC2R 2LS London U.K

**Keywords:** Image reconstruction, positron emission tomography (PET), resolution recovery (RR)

## Abstract

Resolution recovery (RR) techniques in positron emission tomography (PET) imaging aim to mitigate spatial resolution losses and related inaccuracies in quantification by using a model of the system’s point spread function (PSF) during reconstruction or post-processing. However, including PSF modeling in fully 3-D image reconstruction is far from trivial as access to the scanner-specific forward and back-projectors is required, along with access to the 3-D sinogram data. Hence, post-reconstruction RR methods, such as the Richardson–Lucy (RL) algorithm, can be more practical. However, the RL method leads to relatively rapid noise amplification in early image iterations, giving inferior image quality compared to iterates obtained by placing the PSF model in the reconstruction algorithm. We propose a post-reconstruction RR method by synthesizing PET data by a forward projection of an initial real data reconstruction (such reconstructions are usually available via a scanner’s standard reconstruction software). The synthetic PET data are then used to reconstruct an image, but crucially now including a modeled PSF within the system model used during reconstruction. Results from simulations and real data demonstrate the proposed method improves image quality compared to the RL algorithm, whilst avoiding the need for scanner-specific projectors and raw sinogram data (as required by standard PSF modeling within reconstruction).

## I. Introduction

Positron tomography (PET) imaging is a powerful clinical and research tool, one of its major strengths is the ability to provide quantitative values that reflect physiological or biological processes. Hindering that goal is the characteristically low spatial resolution of PET imaging, which leads to inaccurate quantification and degrades image quality [1]. Among other factors, positron range contributes to the loss of spatial resolution in PET. Clinical PET imaging has relied on flourine-18 where the loss of spatial resolution due to the positron range is considered small. Yet, there are an increasing number of radionuclides which emit high-energy positrons that are useful in the clinical and research settings. For example, gallium-68-based radiopharmaceuticals have numerous clinical applications [2], but with a maximum positron range in water approximately fourfold greater than fluorine-18 positron range becomes an important contributor to poor image quality. In low-density tissues, most notably the lungs, positron range increases, and exacerbates the problem [3]. Furthermore, in preclinical small animal imaging, where anatomical structures are more than 100-fold smaller than in humans, positron range can easily be the dominant factor contributing to the deterioration of spatial resolution.

Resolution recovery (RR) techniques attempt to mitigate the loss of spatial resolution. Traditionally, two main techniques have emerged both of which require knowledge of the PET scanner’s point spread function (PSF): 1) incorporating the PSF within statistical iterative algorithms and 2) applying the PSF on a post-reconstruction basis. Contemporary commercial PET scanners often opt for the former approach. Estimating an accurate scanner-specific PSF is nontrivial but several techniques exist and are reviewed elsewhere [4]. Notably, the vendors often use PSF kernels that are based on radionuclides with short positron ranges and model only detector blurring [5]. Building PSF modeling into image reconstruction is far from trivial, to modify such PSF-based image reconstruction to explicitly account for positron range requires access to the forward and back projectors related to the scanner geometry; these are difficult to obtain limiting the application of the technique. Hence, post-reconstruction methods can be far more practical and widely applicable. An example of this latter approach is the Richardson–Lucy (RL) algorithm; a popular implementation in PET imaging [6]. The RL algorithm is simpler to implement since knowledge of the reconstruction algorithm and geometry is not required. Yet, image noise can rapidly accumulate during the process resulting in unacceptably poor image quality. Indeed, noise build-up is an issue for PSF-based reconstructions where early termination and smoothing filters are typically applied as a remedy. More recently, techniques that use artificial intelligence (AI), specifically deep learning, to correct PET images for positron range effects have shown promising results [7]. However, in the context of positron range correction, AI-based methods have yet to be compared to existing techniques, such as PSF-based reconstruction or the RL algorithm.

In this work, we propose a novel post-reconstruction technique in which RR is embedded into a synthesized image reconstruction problem. In essence, this frames the reconstruction task as an inverse problem that favorably decelerates the reconstruction process enabling a better sequence of iterates when an early termination methodology is applied (often the case in practice). Initial findings from a 2-D simulation of a digital thorax phantom demonstrated a performance gain compared to the RL algorithm [8]. Here, we evaluate the method further by investigating the influence of hyperparameters of the synthesized reconstruction algorithm, measure quantitative performance in different sized regions of interest (ROI), compare the approach in additional digital phantoms, and demonstrate the proposed method in real preclinical images.

## II. Method

### A. Synthesized Reconstruction Theory

PET, raw data representing the distribution of a positron-emitting radiotracer measured on a scanner can be conveniently represented in the form of sinograms [9]. The goal of image reconstruction is to model the mean of the PET radiotracer activity concentration distribution that best agrees with a given set of measured coincidences (e.g., sinogram data). This is obtained by maximizing a Poisson log-likelihood objective function; a robust method to obtain the solution is the maximum-likelihood expectation maximization (MLEM) algorithm

\begin{equation*} \boldsymbol {\theta }^{\left ({k + 1 }\right)} = \frac { \boldsymbol {\theta }^{(k)}}{ \boldsymbol {X}^{T} \boldsymbol {1}} \boldsymbol {X}^{T}\frac { \boldsymbol {m}}{ \boldsymbol {X} \boldsymbol {\theta }^{(k)}} \tag{1}\end{equation*}

where $\boldsymbol {X}$ is the system matrix, $\boldsymbol {\theta }^{(k)}$ is the image estimate at iteration k, $\boldsymbol {m}$ are the measured data (e.g., in the form of sinograms), and the denominator $\boldsymbol {X}^{T} \boldsymbol {1}$ is the sensitivity image. The algorithm is typically terminated early to mitigate excessive noise in the final reconstructed image. For PSF-based image reconstruction, resolution modeling can be incorporated via a shift-equivariant PSF kernel contained in a circulant matrix $\boldsymbol {P}$, as follows:

\begin{equation*} \boldsymbol {\theta }^{\left ({k + 1 }\right)} = \frac { \boldsymbol {\theta }^{(k)}}{ \boldsymbol {P}^{T} \boldsymbol {X}^{T} \boldsymbol {1}} \boldsymbol {P}^{T} \boldsymbol {X}^{T}\frac { \boldsymbol {m}}{ {\mathbf {XP}} \boldsymbol {\theta }^{(k)}}. \tag{2}\end{equation*}

This allows the recovery of spatial resolution loss by incorporating knowledge of the PSF into the measurement process. The various components of PSF modeling can be performed in image space [10] or projection space. In practice, measurements of the PSF in PET scanners reveal they are space-variant and anistropic. In this work, the PSF kernel models positron range which is most appropriately modeled in image space [11].

The RL algorithm [12] is a post-reconstruction technique which operates in image space. An update of the RL algorithm is described by

\begin{equation*} \boldsymbol {\theta }^{\left ({l + 1}\right)} = \frac { \boldsymbol {\theta }^{(l)}}{ \boldsymbol {P}^{T} \boldsymbol {1}} \boldsymbol {P}^{T}\frac { \boldsymbol {\theta }^{(K)}}{ \boldsymbol {P} \boldsymbol {\theta }^{(l)}} \tag{3}\end{equation*}

Here, $\boldsymbol {\theta }^{(K)}$ is the reconstructed image using the MLEM algorithm at the final iteration and $K$. $\boldsymbol {\theta }^{(l)}$ is the current image estimate.

In the proposed method, synthetic data, $\boldsymbol {m}_{\mathrm {syn}}$, are generated using a virtual scanner geometry, with a synthesized forward model given by $\boldsymbol {S}$

\begin{equation*} \boldsymbol {m}_{\mathrm {syn}} = \boldsymbol {S} \boldsymbol {\theta }^{(K)}.\tag{4}\end{equation*}

Note that these data are consistent with the system model. PSF modeling is then incorporated via $\boldsymbol {P}$, into a new synthesized image reconstruction problem

\begin{equation*} \boldsymbol {\theta }^{\left ({l + 1 }\right)} = \frac { \boldsymbol {\theta }^{(l)}}{ \boldsymbol {P}^{T} \boldsymbol {S}^{T} \boldsymbol {1}} \boldsymbol {P}^{T} \boldsymbol {S}^{T}\frac { \boldsymbol {m}_{\mathrm {syn}}}{ {\mathbf {SP}} \boldsymbol {\theta }^{(l)}}.\tag{5}\end{equation*}

To summarize, the proposed method takes a reconstructed PET image (which has spatial resolution losses caused by positron range effects not having been modeled) and forward projects the image using a virtual scanner geometry to synthesise sinogram data. These synthetic data are then used in a reconstruction problem with consistent projectors that aims to recover the loss of resolution due to positron range. Importantly, the input image could be obtained from any PET scanner and the system matrix/scanner geometry does not need to be known; on the contrary, the geometry of the virtual scanner can be chosen by the user.

All RR methods require knowledge of the PSF kernel, but notably, MLEM with PSF modeling [see (2)] also requires knowledge of the scanner-specific system matrix, $\boldsymbol {X}$, and the raw measured data, $\boldsymbol {m}$. Despite apparent similarities between the proposed method and MLEM+PSF [i.e., comparing (2) and (5)], the proposed method only requires an image, $\boldsymbol {\theta }^{(k)}$, and the matrix $\boldsymbol {P}$ in order to perform RR. This has a significant practical advantage, whilst offering the potential for performance comparable to the characteristics of MLEM+PSF (which is favorable to the RL method in the majority of practical use cases).

### B. Data Simulation

2-D digital phantoms representing three different count levels (high, mid, and low) were simulated and taken as the ground truth. These simulations represent PET acquisitions, which for example correspond to differences in injected activity or acquisition times. The modified Sheep-Logan [13], BrainWeb [14], and Zubal thorax phantoms [15] were used. To assess the spatial resolution, a 2-D slice of a Derenzo-style microPET phantom was simulated, the rod diameters were 1.1, 1.5, 2.3, 3.1, 3.9 and 4.7 mm; a ratio of 4:1 was used for the rod to background radioactivity concentration. A 2-D Gaussian function was used to model the PSF for a gallium-68-based radiopharmaceutical; the PSF represents the contribution of positron range alone with a full width at half maximum (FWHM) of 2.9 mm [16]. The measured data were simulated as follows: 1) ground truth 2-D images were blurred using the PSF model for gallium-68; 2) blurred images were forward projected using a parallel line integral model (the Radon tranform) into a sinogram with angles in the range 1° to 180° at 1° intervals; and 3) random Poisson noise was added to the sinogram.

The proposed method was compared to the RL algorithm, MLEM without and with PSF modeling, defined by (1) and (2), respectively; herein, they are referred to as MLEM and MLEM+PSF. MLEM+PSF is regarded as the reference standard in this work.

For the MLEM algorithm, the system matrix, $\boldsymbol {X}$, was the Radon transform with projection angles in the range 1° to 180° at 1° intervals. The input image for the RL algorithm and the proposed method was reconstructed by MLEM $(\boldsymbol {\theta }^{(K)} \boldsymbol {)}$. Unless otherwise stated, the value of ${K}\,\,=$ 64, this value was based on the experience of a typical clinical reconstruction software setting.

For the proposed method, the system matrix, $\boldsymbol {S}$, was a discrete Radon transform. Unless otherwise stated the projection angles were in the range 1° to 180° at 1° intervals.

For all methods, the number of iterations chosen was large enough to ensure convergence (typically several hundred were required; depending amongst other factors on the phantom and count level).

MATLAB version R2020b [17] was used to implement the reconstruction algorithms and perform data analyses.

### C. Experimental Preclinical Data

The proposed method was qualitatively assessed on experimental preclinical data. ^68^Ga-THP-PAM is a bone-seeking radiopharmaceutical which accumulates in the oxyhaptite crystals of bone. The imaging protocol is described in detail elsewhere [18], here we summarize the details. Images were acquired using the Mediso Nanoscan PET/CT with attenuation and scatter correction. Approximately 1.8 MBq was intravenously injected into the tail vein of a immunocomprised mouse and data were acquired for 60 min. Images were reconstructed using MLEM with 60 iterations with an isotropic voxel size of 0.21 mm. The number of projection angles were between 1° and 180° at 1° intervals. Given the preclinical data represents blurring due to the positron range in 3-D, we modeled the PSF kernel as a 3-D Gaussian function, but implemented the forward and back-projectors in 2-D. To assess whether a more accurate PSF model for the positron range would be benefical, we implemented a monoexponential function to model the PSF. Previous studies [19] have shown that the positron range of gallium-68 in high-resolution PET can be well modeled as a monoexponential with an attenuation coefficient of 0.77 mm^−1^.

### D. Image Evaluation

The normalized root mean square error (RMSE) was the metric used to evaluate quantitative agreement with the ground truth. RMSE can be defined in terms of the bias and standard deviation of the image; it was used to compare the performance of the different methods

\begin{equation*} {\mathrm{ RMSE = }}\sqrt {\mathrm {Bias}^{2}+\mathrm {StdDev}^{2}}.\tag{6}\end{equation*}

With the bias and standard deviation defined by

\begin{align*} \mathrm {Bias}=&\sqrt {\frac {\sum _{j \in \boldsymbol {\Omega }}\left ({{\overline { \boldsymbol {\theta }}}_{j}^{(k)}- \boldsymbol {\theta }_{j}^{{\mathrm {ref}}} }\right)^{2}}{\sum _{j \in \boldsymbol {\Omega }}\left ({\boldsymbol {\theta }_{j}^{{\mathrm {ref}}} }\right)^{2}}} \tag{7}\\ \mathbf {StdDev}=&\sqrt {\frac {1}{Q}\frac {\sum _{q = 1}^{Q}{\sum _{j \in \boldsymbol {\Omega }}\left ({{\overline { \boldsymbol {\theta }}}_{j}^{(k)}- \boldsymbol {\theta }_{j}^{\left ({{q,k} }\right)} }\right)^{2}}}{\sum _{j \in \boldsymbol {\Omega }}\left ({\boldsymbol {\theta }_{j}^{{\mathrm {ref}}} }\right)^{2}}} \tag{8}\end{align*}

where ${Q}$ is the number of noise realizations, $\boldsymbol {\theta }_{j}^{{\mathrm {ref}}}$ are the pixel values of the true object, and ${\overline { \boldsymbol {\theta }}}_{j}^{(k)}$ is the mean reconstructed value for pixel ${j,}$ at iteration ${k}$. We evaluated these metrics globally (i.e., ${\Omega }\,\,=$ entire image), and in a medium-sized ROI (70 pixels) and a small ROI (6 pixels) for features of interest. The number of noise realizations were chosen to ensure results were statistically valid and varied depending on the size of the domain: for global and medium ROI ${Q}\,\,=$ 10 and for the small ROI ${Q}\,\,=$ 100.

## III. Results

### A. Qualitative Comparison and RMSE

**Fig. 1.**
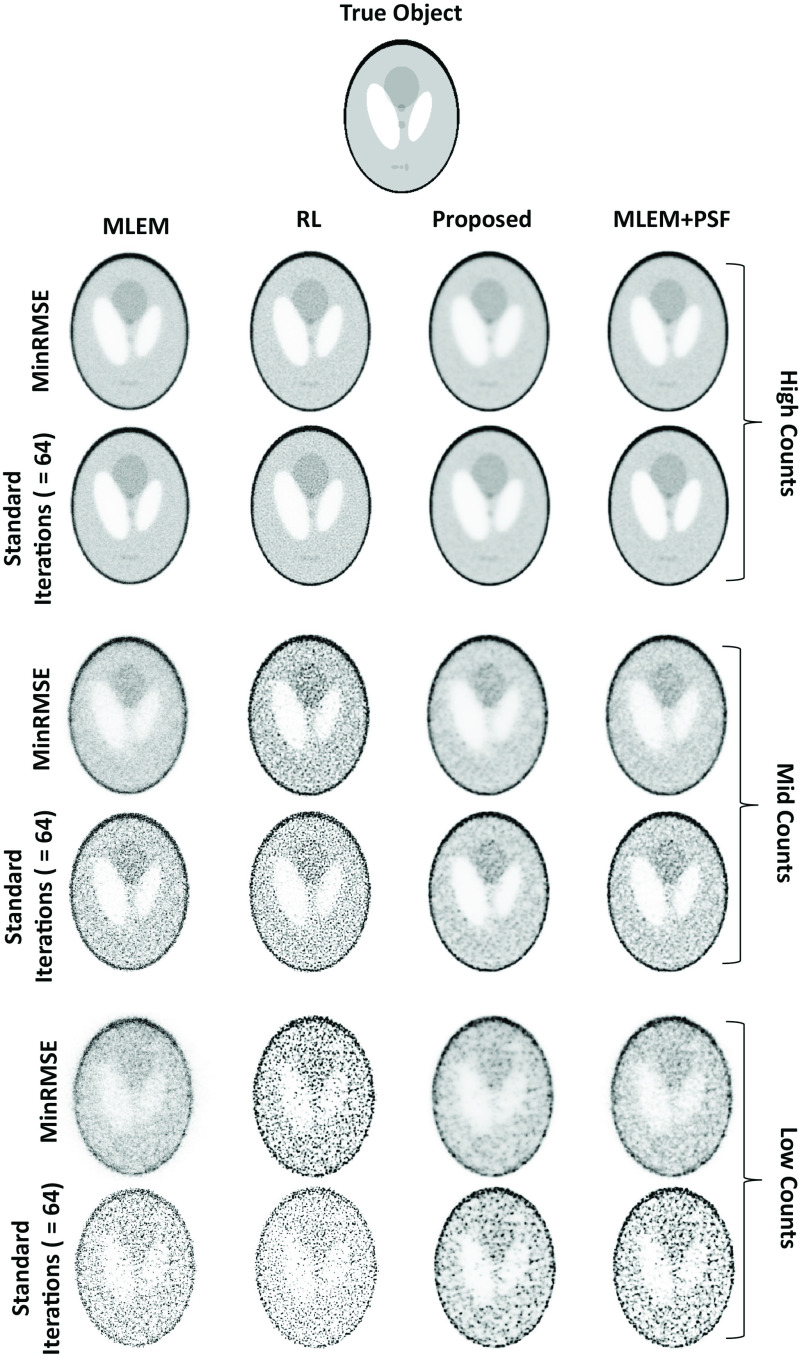
RR methods at different count simulations for the modified Shepp–Logan phantom. The true object is shown at the top. For each count simulation (either high, mid, or low) the top row corresponds to images obtained at the minimum RMSE, the bottom row corresponds to the images at 64 iterations.

**Fig. 2.**
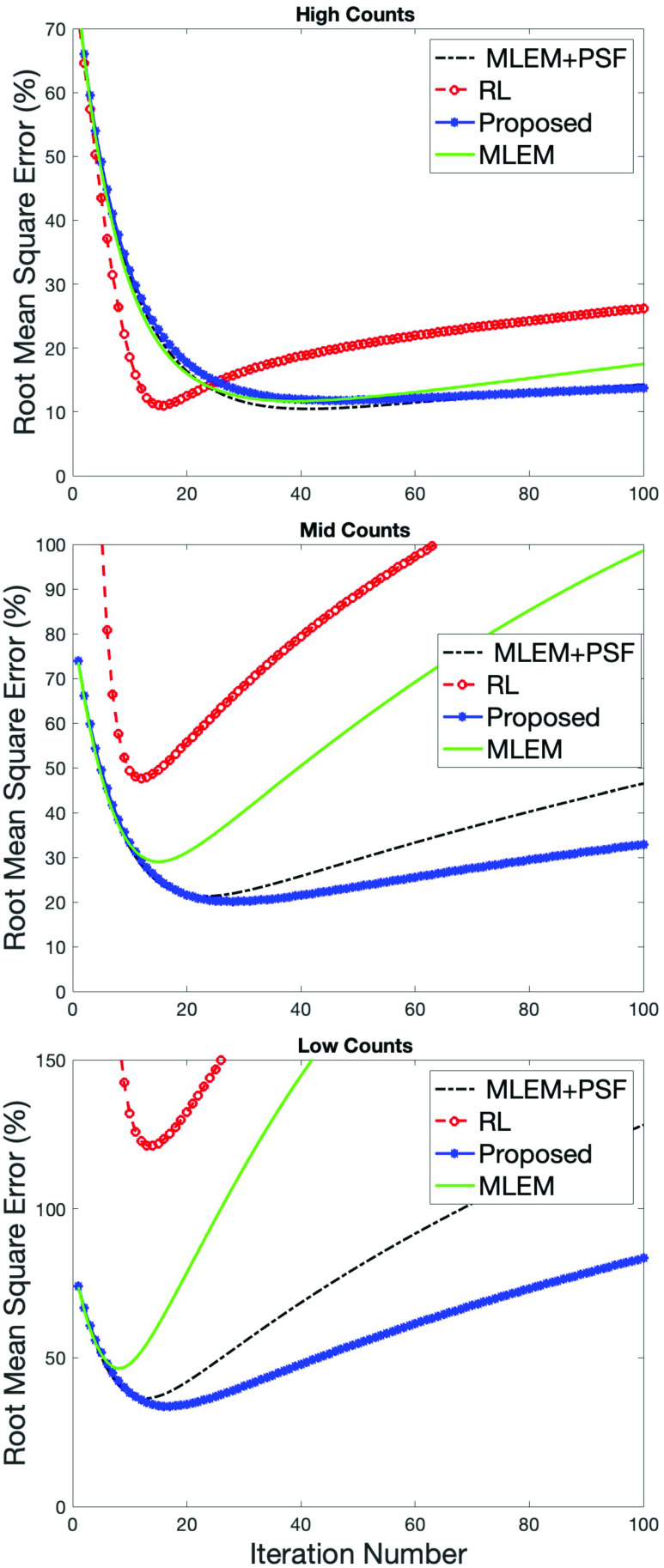
Normalised RMSE as a function of number of iterations using various methods at different count levels for the Shepp–Logan phantom. Upper row corresponds to high-count image, middle row to a mid-count image, and bottom row to a low-count image. As the count level decreases the proposed method exhibits a relative performance gain compared to the RL algorithm.

Fig. 1 shows reconstructed images for the modified Shepp–Logan digital phantom at three different simulated count levels. For each simulated count level, the top row (“MinRMSE”) corresponds to the minimum RMSE achieved for each method, i.e., the best agreement with the true object. Outside of simulated images the true object is unknown; hence, the bottom row shows a more realistic case obtained after a “standard number” of iterations (= 64), typical of a clinical scanning scenario. The minimum RMSE images obtained using the RL algorithm are characterized by a noisy appearance in the lower count regimes, more so for the standard iteration images. Comparatively, the proposed method has produced images which have mitigated the noise. In fact, the proposed method has produced images that are very similar in appearance to MLEM+PSF, our reference method for RR. Indeed, the plots of RMSE as a function of iteration number reflect this observation (see Fig. 2). At high counts, all the methods achieve a comparably low minimum RMSE; albeit the RL algorithm converges in the fewest iterations. However, with increasing iterations the RMSE for the RL algorithm increases compared to the other techniques as noise begins to dominate. For mid and low-count simulations, the performance gains of the proposed method become clear. For the RL algorithm, there is a rapid deterioriation in the minimum RMSE achieved (e.g., low counts: 124% for RL versus 33% for proposed), with the proposed method exhibiting less variation in RMSE with an increasing number of iterations. Fig. 2 also demonstrates a surprising observation: the similarity of the proposed method to MLEM+PSF (the reference method). Indeed, at low counts there may be a slight performance gain using the proposed method over MLEM+PSF. The proposed method does not have access to the measured sinogram of the true object yet is able to achieve comparable performance to MLEM+PSF. These findings were replicated across all phantoms (see our initial findings in the Zubal thorax [8]).

**Fig. 3.**
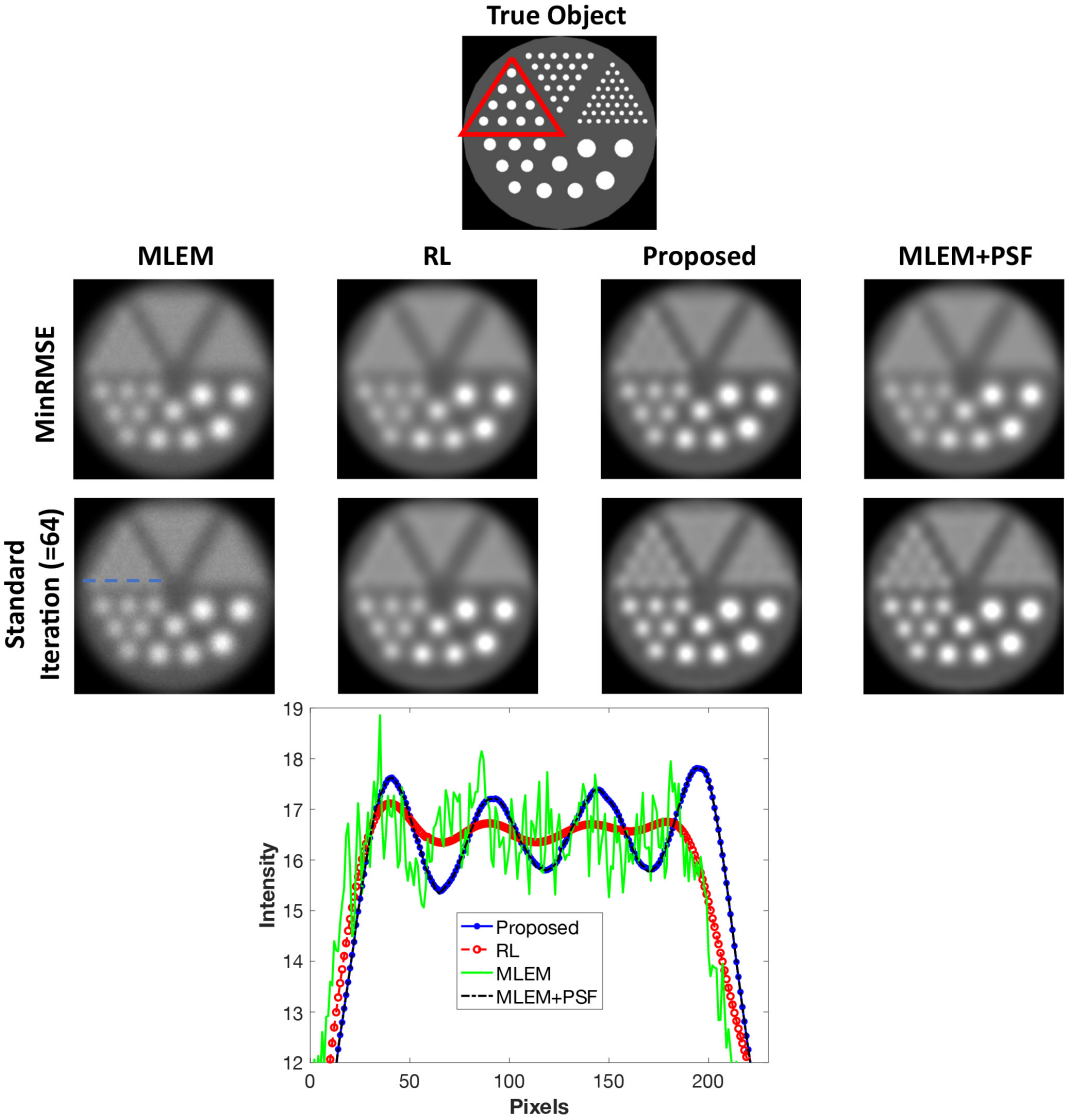
RR methods applied to a simulated Derenzo phantom at the mid-count level. The true object is shown at the top of the image, the red triangle indicates the 2.3-mm diameter rods. The first row represents images obtained at the minimum RMSE, the second row represents images at 64 iterations. The 2.3-mm rods are more distinguishable using the proposed method and MLEM+PSF than the RL algorithm. At the bottom, line profiles (dashed blue line is indicative of position) show the improvement of the proposed method compared to the RL method on standard iteration reconstructions.

Fig. 3 shows the results of the simulation of the Derenzo phantom at the mid-count level. The true object is shown at the top of the figure for reference. None of the methods were able to resolve the two smallest diameter rods at any count level. Although RR was achieved for the largest rods with all the applied methods, only for the proposed method and MLEM+PSF is there a noticable improvement in the 2.3-mm rods (highlighted in red on the true object). This behavior is repeated for the low-count simulations; at high counts the methods performed similarly.

A unique feature of the proposed method is the virtual scanner geometry defined by the matrix, $\boldsymbol {S}$, in (4). Consequently, the performance of the proposed method will depend on hyperparameters related to $\boldsymbol {S}$ and the initial quality of the reconstructed image.

### B. Synthetic Projection Angles

**Fig. 4.**
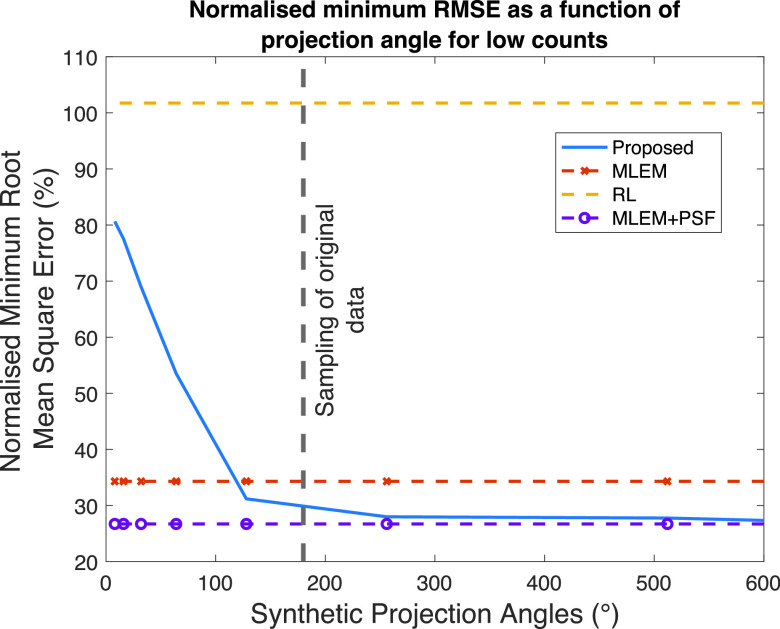
Minimum RMSE as a function of the number of projection angles in the synthetic geometry for the Zubal thorax phantom at low counts. For reference, the minimum RMSE is shown for the comparison methods.

Within the synthetic geometry, we varied the number of projection angles defined within system matrix $\boldsymbol {S}$ [note this also affects the synthesized data, $\boldsymbol {m}_{syn}$, in (3)]. Fig. 4 shows the minimum RMSE achieved using the proposed method when varying the projection angles for the Zubal thorax phantom at a low-count acquisition. Values on the $x$-axis indicate sampling every 1° until that value, e.g., a value of 100° = between 1° and 100° in 1° increments. The input image to the proposed method was an MLEM reconstruction terminated at 64 iterations and was performed with 1° angular sampling between 1° and 180° (indicated by native sampling in Fig. 3).

For reference, the minimum RMSE achieved by the comparison methods are shown; these methods have no dependence on synthetic projection angles. Performance degrades when using fewer projection angles than the supplied MLEM reconstruction. There appears no improvement in RMSE beyond the original sampling used in the input image (in this case 180°); This behavior is consistent across different count acquisitions and phantoms.

### C. Line Thickness

**Fig. 5.**
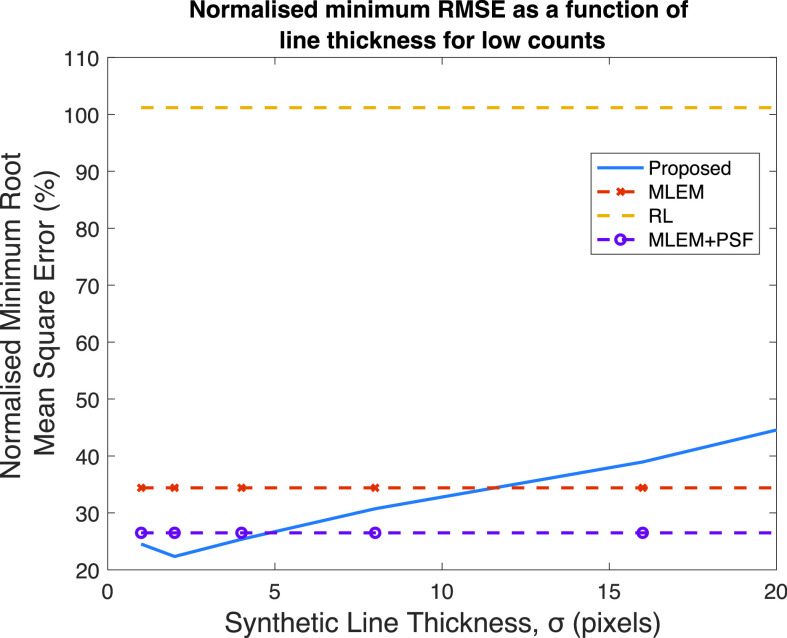
Minimum RMSE as a function of line thickness in synthesized reconstruction for the Zubal thorax phantom at low counts. The comparison methods are shown for reference.

The virtual geometry was also modified by applying a kernel which varies the thickness of the line integral in the forward projection of the synthetic reconstruction. This was achieved by applying a spatially invariant 2-D Gaussian function to the input image, $\boldsymbol {\theta }^{(K)}$ (from the MLEM algorithm with 64 iterations). The hyperparameter in this case is ${\sigma }$ from the Gaussian function. The 2-D Gaussian function was then modeled in the synthetic reconstruction to recover this introduced blur. Fig. 5 shows the minimum RMSE achieved as a function of ${\sigma }$ for the Zubal thorax phantom at low counts. The synthetic projection angles were between 1° and 180°, matching the angular sampling used in the MLEM reconstruction used as the input image to the proposed method. As a reference the minimum RMSE achieved with the RL, MLEM, and MLEM+PSF algorithms are shown. For low-count data in the Zubal thorax phantom, there is a slight performance improvement when increasing the line thickness at the level of ${\sigma } = 2$ pixels, increasing the value of ${\sigma }$ then results in a deterioration of performance. No performance gain was observed in the mid and high-count images.

### D. Number of Iterations of Supplied MLEM Reconstruction

The performance of the proposed method and the RL algorithm will depend on the input image; another hyperparameter is the number of iterations, ${K}$, used in the MLEM reconstruction that supplies the input image.

**Fig. 6.**
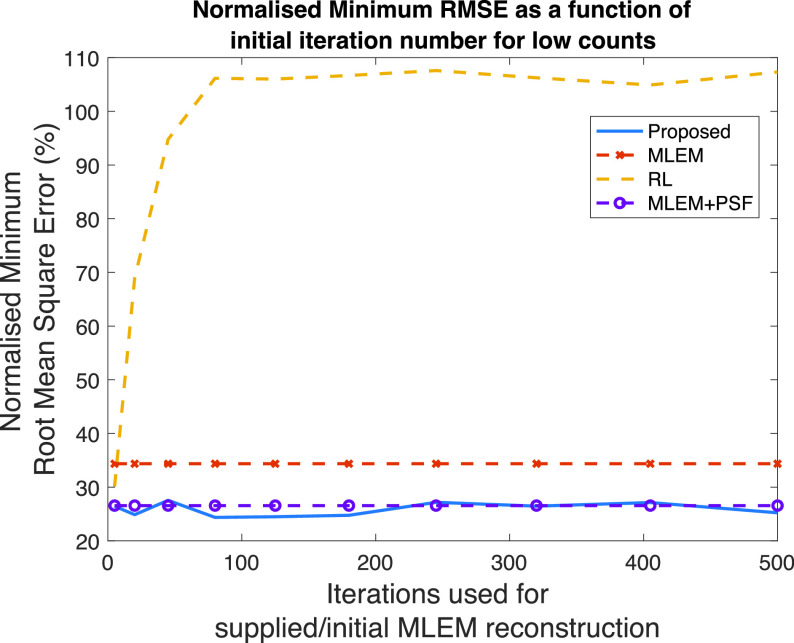
Minimum RMSE as a function of iterations used in the supplied MLEM reconstruction. Both the proposed method and the RL algorithm varying with input image quality. MLEM and MLEM+PSF are shown for reference.

Fig. 6 shows the minimum RMSE as a function of the number of iterations of the supplied MLEM input image for the Zubal thorax phantom at low counts. For reference, the minimum RMSE achieved using MLEM and MLEM+PSF reconstructions are shown; clearly these will not depend on an input image, as they operate on the sinogram data. The minimum RMSE for the RL algorithm rapidly increases between 5 and 100 iterations of the MLEM algorithm. In contrast, the proposed method is less dependent on the image quality, even when operating on relatively poor-quality input images, a low minimum RMSE is achieved. This pattern of favorable performance holds for the different count simulations: with the high-count simulation, the increase in minimum RMSE for the RL algorithm is less steep but, the proposed method has more stable dependence on the input image.

### E. Variation on the Proposed Method and Bias/Standard Deviation Tradeoff

Earlier we hypothesized that the data, $\boldsymbol {m}_{{\mathrm {syn}}}$, being consistent with the reconstruction problem would, at least partly, confer a benefit over the usual reconstruction problem with inconsistent measured projection data. We applied the synthesized reconstruction method without PSF modeling; equivalent to (5) without PSF kernel matrix, $\boldsymbol {P}$, to investigate if there is a performance gain compared to MLEM, RL, or MLEM+PSF algorithms.

**Fig. 7.**
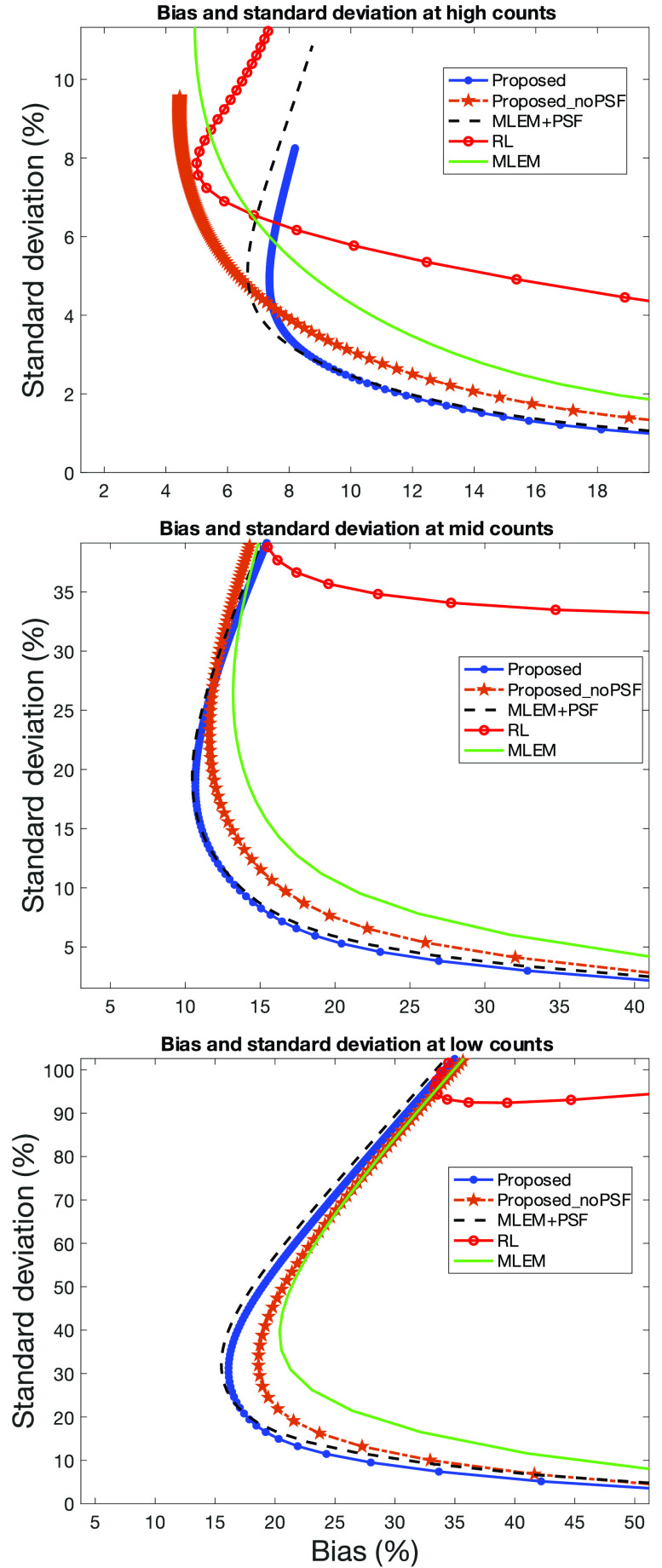
Global bias and standard deviation for Zubal Phantom at different count simulations. The graph at the top corresponds to the high-count simulation; middle is the mid count and bottom is low count.

In Fig. 7, the global bias and standard deviation tradeoff is shown for the proposed method, the variation without PSF modeling (displayed as Proposed_noPSF), and the comparison methods for Zubal thorax phantom with different simulated count statistics. In the high-count simulations, the RL algorithm achieves a low bias but at increased variance compared to MLEM+PSF and the proposed method. For lower count simulations, the RL algorithm results in increasing bias and variance. In contrast, the proposed method achieves a low bias and standard deviation across different count regimes; Fig. 7 demonstrates that the MLEM+PSF algorithm produces the most desirable bias and standard deviation. However, the similarity to the proposed method is clear. The proposed method without PSF modeling exhibits improved performance over MLEM and the RL algorithm despite not modeling the PSF; this is more evident in the low-count simulations.

**Fig. 8.**
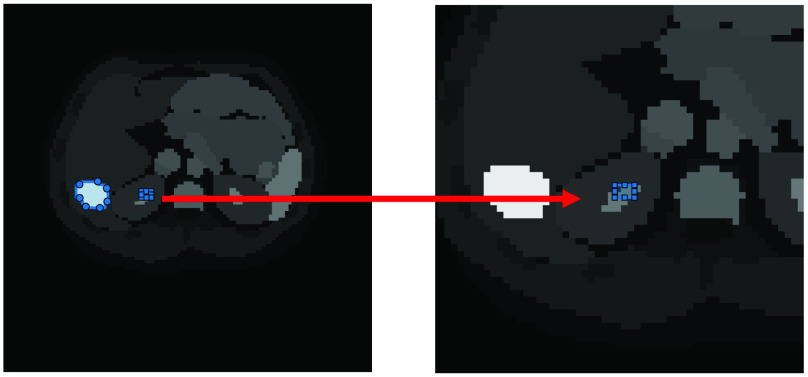
ROI definitions for the Zubal Phantom. A medium ROI of 60 pixels was delineated around the high contrast region indicated and a small ROI of 6 pixels was delineated adjacent.

**Fig. 9.**
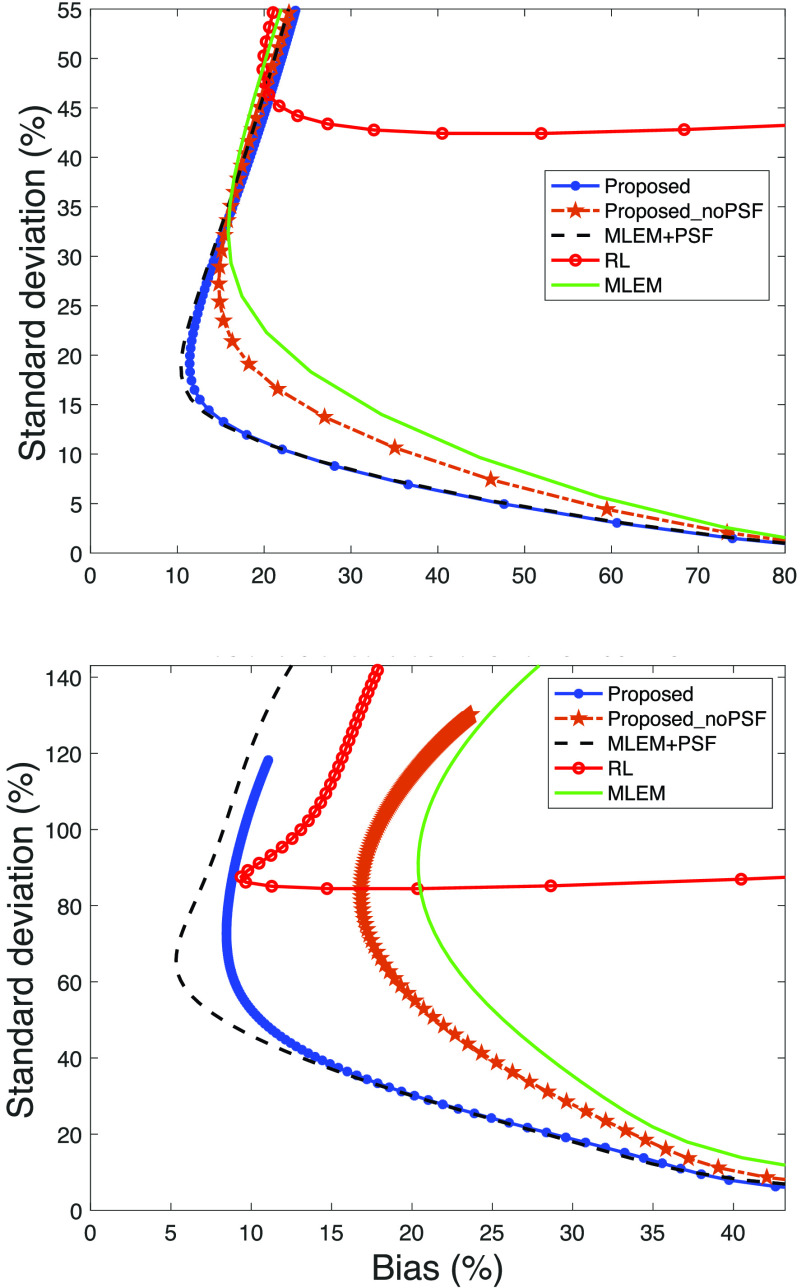
Bias and standard deviation for low-count simulations in a medium (top) and small ROI (bottom) of the Zubal thorax phantom.

Besides the global values of bias and standard deviation, we calculated the values for a medium and small ROI in the Zubal thorax phantom (ROIs defined in Fig. 8). Fig. 9 shows the results in the low-count simulation. In agreement with the global values, in the medium-sized ROI the proposed method achieves a lower bias and standard deviation compared with the RL algorithm; similarly, the proposed method without PSF modeling achieves a performance gain albeit less marked. Consistent with previous findings the MLEM+PSF algorithm exhibits the most desirable behavior. Nevertheless, the proposed method is strikingly similar to MLEM+PSF.

For the small ROI, the relative difference between the RL algorithm and the proposed method is less distinct. The RL algorithm demonstrates a similar bias to the proposed method but still at the cost of increased variance. The proposed method without PSF modeling produces improved values compared to MLEM.

### F. Experimental Results

**Fig. 10.**
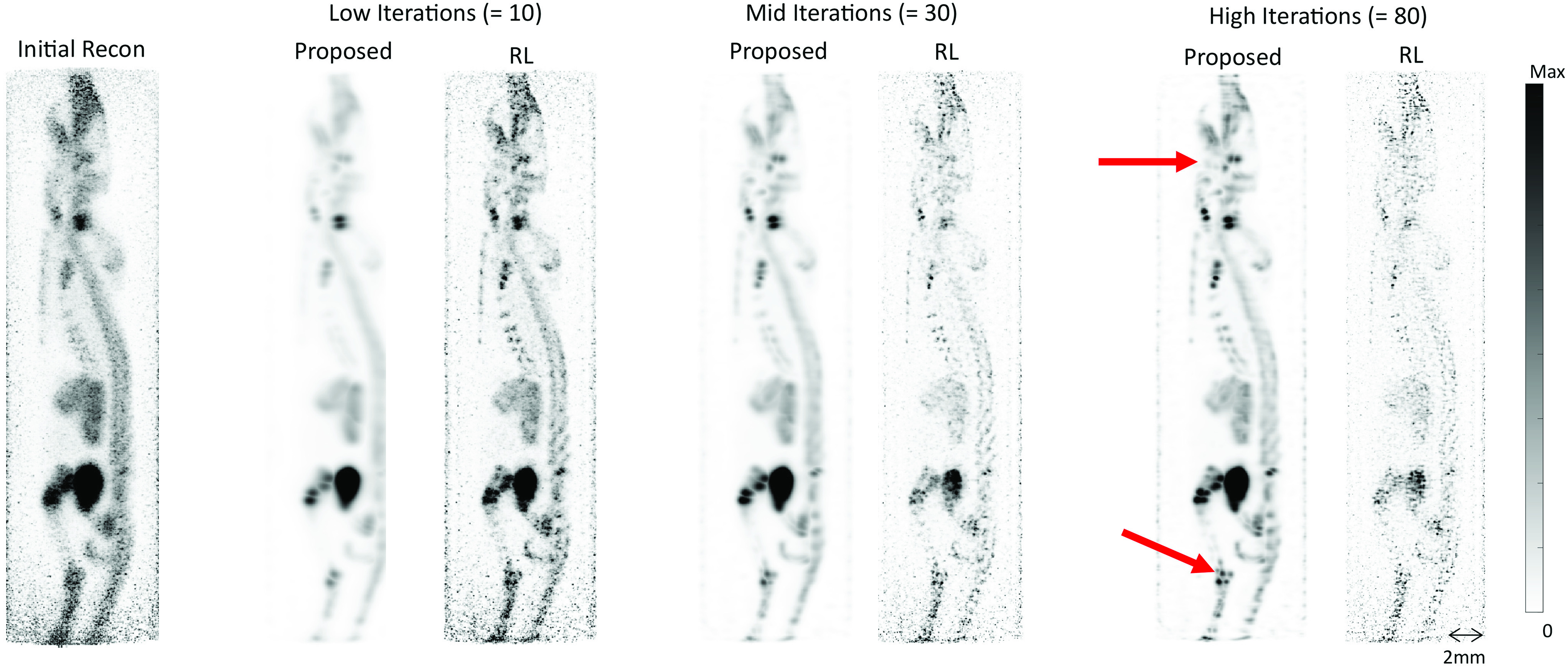
RR on preclinical images at different iterations. Images are sagittal MIPs. The input image is labeled as Initial Recon; the proposed method is compared to the RL algorithm. Details are more clearly resolved (examples indicated by red arrows) using the proposed method.

To assess whether the benefits of the proposed method translate beyond simulations, we applied the technique to experimentally acquired preclinical data. Representative maximum intensity projection (MIP) images of ^68^Ga-THP-PAM in a mouse are shown in Fig. 10. The initial reconstructed images were obtained using a standard preclinical protocol with the native reconstruction software. The RL algorithm and proposed method are shown at low, mid, and high iterations. Several features are more readily resolved using the proposed method. Although the RL algorithm does recover resolution from the initial reconstruction it is more severely affected by noise making certain details more difficult to resolve.

**Fig. 11.**
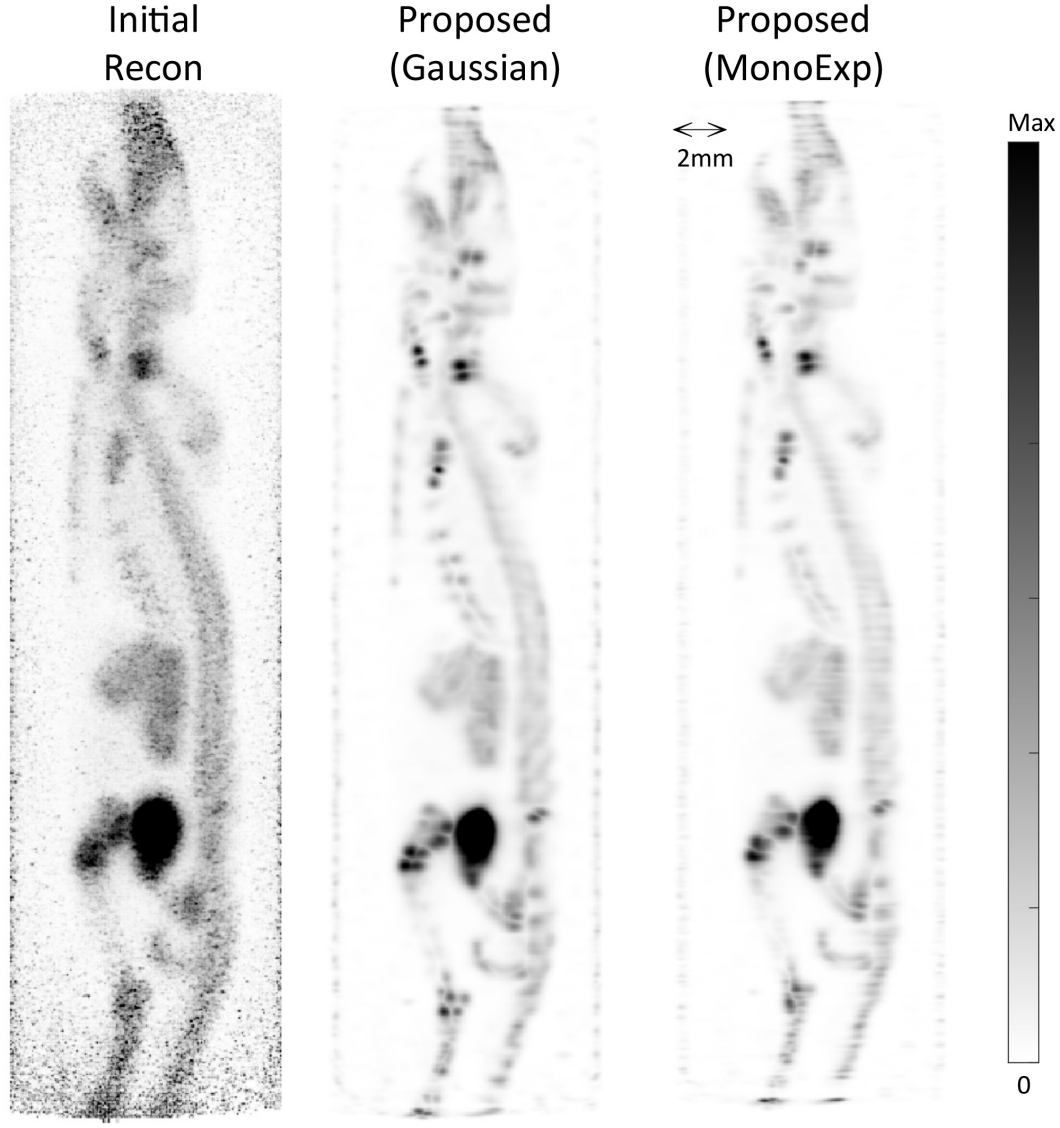
RR on preclinical images with alternative positron range modeling. Images are sagittal MIPs. The input image is labeled as Initial Recon. MIP using the proposed method with a Gaussian function modeling positron range at 80 iterations (same as in Fig. 10.) is shown in the middle. On the right is the MIP at 80 iterations using the proposed method with a monoexponential function to model the positron range. RR is clearly shown using this alternative model.

Fig. 11 shows the results of the proposed method with different PSF models, a Gaussian and a more realistic monoexponential, using the same mouse data as Fig. 10. Based on the results in Fig. 10 the number of iterations was chosen as 80 for the proposed method. Comparing the initial reconstruction with no resolution modeling to the proposed method using a monoexponential model it is clear that the resolution has been improved. Importantly, the monoexponetial PSF model also yields the same improvement in noise control as the Gaussian PSF model when compared to the RL algorithm.

## IV. Discussion

We have proposed a post-reconstruction RR method based on synthesized image reconstruction. To summarize the method: first, sinogram data are synthesized from a real data reconstruction using a virtual scanner; and second, an image is reconstructed using the synthesized sinogram and virtual scanner’s system matrix. Importantly, the synthetic reconstruction incorporates PSF modeling which allows RR of the original real data reconstruction. We demonstrated the technique using 2-D PET data by modeling the degradation of spatial resolution due to the positron range. Besides outperforming the widely used RL algorithm both in terms of quantitative and qualitative metrics, our findings show comparable performance to PSF-based reconstruction. Although these promising findings are limited to recovery of positron range resolution losses, the flexibility of the method would allow it to be extended to other resolution degrading effects and even other medical imaging modalites.

In this work, we benchmarked the performance of our method against the RL algorithm; given it is a well-known post-reconstruction RR method in medical imaging. In high-count simulated PET images, the RL algorithm and proposed method achieved similar minimum RMSE values and comparable image quality. However, the proposed method delivered a substantial relative performance improvement in the mid and low-count simulations. The RL algorithm produced images that were noisy and of poorer image quality than the proposed method. Indeed, this was reflected in the higher global variance of images produced by the RL algorithm. Simulation of a Derenzo phantom also revealed improvements in spatial resolution using the proposed method. Although ideally one would acquire a high number of counts in every acquisition, in practice the requirement to minimis radiation dose and scanning times mean the lower count simulations may be more realistic in many real clinical and research settings. Moreover, there are several promising radiotracers, particularly for therapeutic applications, that have a low branching ratio for positron decay [20], yielding poor count statistic images.

Beyond simulations, when using either the RL algorithm or the proposed method for RR, choosing the optimal iteration number is a challenge (since the ground truth is unknown). Yet, Fig. 2 reveals a potential additional benefit to the proposed method over the RL algorithm: a slower increase in RMSE as a function of a number of iterations. Hence, over-iterating will have less impact on image quality than for the RL algorithm. Among other factors post-reconstruction RR will be dependent on the quality of the initial reconstruction (i.e., the input image). Our results suggest that the proposed method is less dependent on a number of iterations of the initial reconstruction (taken as a surrogate of image quality) than the RL algorithm.

Our reference standard for RR was PSF-based reconstruction, specifically, the incorporation of the PSF kernel into an MLEM algorithm (MLEM+PSF). Evidently, there are similarities between the proposed method and MLEM+PSF [e.g., compare (2) and (5)]; however, there are subtle yet important distinctions. PSF-based reconstruction can be challenging to implement as it requires access to proprietary information; specifically, the forward and back projectors of the scanner. A benefit of the proposed method is that it can be applied without such knowledge, hence can be far more practical and widely applicable. In addition, the proposed method does not require access to the raw measured sinogram data needed for the PSF-based reconstruction. Although RR using PSF-based reconstruction yielded the best performance overall, the proposed method was often comparable; this was apparent both visually and quantitatively. In short, the proposed method has the benefits of a post-reconstruction methodology yet demonstrated comparable performance to a PSF-based reconstruction approach to RR.

A unique aspect of the proposed method is the virtual scanner geometry defined by $\boldsymbol {S}$. In principle, an investigator is able to choose any synthetic geometry. We explored the impact of the hyperparameters of the synthesized reconstruction. Our findings suggest for parallel line projectors, there is a little additional benefit to altering the angular sampling from that used to acquire the original data. Equally, increasing the thickness of the line integral (defined by ${\sigma }$) yielded only a modest improvement in minimum RMSE, but in most cases increasing ${\sigma }$ led to poorer performance. However, it is important to recognize that these values of ${\sigma }$ represent extreme levels of image blurring—chosen to test the limits of the method. Additional work is required to identify the optimal geometry and its relative importance compared to the introduced resolution modeling via $\boldsymbol {P}$.

We also created a synthesized reconstruction problem without PSF modeling and found that the bias and variance of the resulting reconstructed image was improved compared to the original MLEM image. An interesting comparison exists to nested-EM techniques: in previous work it has been shown that interleaving a standard MLEM update with an RL iteration accelerates convergence at the cost of increased variance [21]. In contrast, our proposed method could be considered as a de-nesting of the problem, by removing resolution modeling and performing it in its own synthesized inverse problem. Indeed, we observed a decelerated convergence with performance gains.

The simulated results demonstrate the relative performance gains using the proposed method. However, in the simulated results, we primarily used the same forward and back-projectors for the synthetic reconstruction as the original MLEM reconstruction. In practice, it is unlikely we would have access to these projectors for a commercial PET scanner. Nevertheless, the anecdotal real preclinical data illustrate that the technique can be applied to an unknown geometry with improvements over the RL algorithm. In this work we have modeled only contributions due to positron range in the PSF kernel, but there are numerous other factors which contribute to the loss of spatial resolution in PET; additional considerations are needed to extend the method to account for these. Contemporary commercial scanners often include resolution modeling within their reconstruction software to account for some of these effects; however, correction for the positron range is not typically included. Moreover, positron range is an important contributor to resolution loss in preclinical imaging [20], in clinical imaging of high energy positron emitters [22], [23], and in tissues with low densities such as the lungs [3]. We also demonstrated that the proposed method improves resolution of real data using a more accurate PSF-model (a monoexpontential). However, further modifications could include using a spatially variant tissue-specific model since the positron range distribution depends on the tissue. In this work, we did not include regularization within the synthesized reconstruction, it is possible this could further improve the image quality, particularly the bias and standard deviation tradeoff, of the synthesized reconstruction. Recently deep neural networks have been used to correct PET images for positron range [7]. The authors demonstrated improved noise characteristics and RR compared to PET images with no RR. Comparison of the proposed method to AI-based methods is outside the scope of this present work but could be explored in future studies. We demonstrated the feasibility of the technique in 2-D data only; further work would be required to determine if these benefits extend in a 3-D implementation of the synthetic projectors.

The proposed method is a versatile technique and has many potential applications outside of PET imaging. As a post-reconstruction technique it circumvents some of the major limitations of PSF-based RR whilst mitigating the build up of noise common in the RL algorithm. We implemented only one variation of the approach (removing PSF modeling). However, we note several other potential modifications that may offer improvements in domains where the RL algorithm is commonplace; for example, in astronomical images where the nontomographic data could be synthetically reconstructed with a virtual scanner.

## V. Conclusion

We proposed a novel post-reconstruction RR method using a synthesized image reconstruction framework. As a post-reconstruction technique, the method is potentially more practical and widely applicable than building resolution modeling into image reconstruction, which is challenging without knowledge of the forward and back projectors of the PET scanner. In a variety of digital phantoms and in real preclinical PET data the method outperformed the RL algorithm—a widely used post-reconstruction RR method. The relative performance gains increased with lower count acquisitions. Remarkably the RMSE and image quality were comparable to PSF-based MLEM reconstruction despite the method having no access to the original measured sinogram data.
